# Inactivation of *sacB* Gene Allows Higher 2,3-Butanediol Production by *Bacillus licheniformis* from Inulin

**DOI:** 10.3390/ijms252211983

**Published:** 2024-11-07

**Authors:** Emanoel Gergov, Penka Petrova, Alexander Arsov, Ina Ignatova, Lidia Tsigoriyna, Nadya Armenova, Kaloyan Petrov

**Affiliations:** 1 Institute of Microbiology, Bulgarian Academy of Sciences, 1113 Sofia, Bulgaria; emanoelgergov@microbio.bas.bg (E.G.); ppetrova@microbio.bas.bg (P.P.); al.arsov@microbio.bas.bg (A.A.); 2 Institute of Chemical Engineering, Bulgarian Academy of Sciences, 1113 Sofia, Bulgaria; ina.ignatova@iche.bas.bg (I.I.); lidinka29@gmail.com (L.T.); nadq.armenova@gmail.com (N.A.)

**Keywords:** 2,3-butanediol, *Bacillus licheniformis*, *sacB*, exopolysaccharides, inulin

## Abstract

*Bacillus licheniformis* 24 (BL24) is an efficient, non-pathogenic producer of 2,3-butanediol (2,3-BD). However, during inulin fermentation, the strain produces large amounts of exopolysaccharides (EPS), which interfere with the process’ performance. The present study aims to investigate the effect that inactivation of the *sacB* gene, encoding levansucrase in BL24, has on 2,3-BD production efficiency. Knockout of the *sacB* gene was accomplished via insertional inactivation. The *sacB*-knockout variant formed 0.57 g/L EPS from sucrose and 0.7–0.8 g/L EPS from glucose and fructose, a 15- and 2.5-fold reduction relative to the wild type, respectively. Likewise, during batch fermentation with soluble inulin Frutafit^®^ CLR, the mutant BLΔ*sacB* produced significantly less EPS than the wild type, allowing the maintenance of pH at values favoring 2,3-BD synthesis. At pH 6.50, BLΔ*sacB* reached a record titer of 128.7 g/L 2,3-BD, with productivity of 1.65 g/L/h, and a yield of 85.8% of the theoretical maximum. The obtained concentration of 2,3-BD is two-fold higher compared to that of the wild type. Subsequent RT-qPCR assays confirmed a successful *sacB* knockout. Three of the genes involved in inulin hydrolysis (*sacA*, *sacC*, and *fruA*) maintained their expression levels compared to the wild type, while that of *levB* increased. Although total EPS accumulation could not be completely eliminated via *sacB* gene knockout alone, the overall reduction in EPS content has enabled the highest yield of 2,3-BD from inulin to date, a promising result for the industrial production from inulin-rich substrates.

## 1. Introduction

*Bacillus licheniformis* is an unpretentious soil bacterium with wide and varied industrial applications. As a Gram-positive, spore-forming, and non-pathogenic species, *B. licheniformis* attracted great biotechnological interest as a microbial factory for the synthesis of enzymes (protease, keratinase, amylase, cellulase, allantoinase, chitinase, arabinase, levanase, etc.), bioactive compounds (lipopeptides), exopolysaccharides (EPS), polyhydroxyalkanoates, and hydrogen [1,2,3,4]. Particularly important, however, is the application of the species in obtaining 2,3-butanediol (2,3-BD), a greatly demanded platform chemical for butadiene, methyl ethyl ketone, and polyurethane maleamide production, often used as an antifreeze agent and fuel additive [5,6]. Currently, 2,3-BD is produced by chemical synthesis from petroleum. The microbial production of 2,3-BD is considered white biotechnology as it replaces the chemical synthesis of an industrially important reagent with a microbial one, with the possibility of utilizing renewable substrates, including cellulose and lignocellulosic materials, molasses, food waste, and inulin [7,8,9,10,11,12]. Due to rising oil prices, pilot plants were built in the United States and China to process pretreated lignocellulosic waste into 2,3-BD. Despite recent scientific progress, industrial microbial production of 2,3-BD has yet to be launched [13].

The highest productivity of 2,3-BD was traditionally reported in *Klebsiella pneumoniae* and *K. oxytoca* [14,15], both pathogenic bacterial species. However, considering the titer and yield from glucose, they are already equaled by GRAS (Generally Regarded As Safe) producers of the genera *Penibacillus* and *Bacillus* [16,17]. Among them, *B. licheniformis* is preferred, as it can achieve a high concentration of 2,3-BD (up to 150 g/L) and a yield close to the theoretical maximum (0.44–0.47 g/g), both from glucose and fructose [13,18]. Furthermore, this species is also capable of converting inulin-containing feedstocks, which is promising for the profitability of the potential industrial process [19].

Inulin is a poly-fructan, abundant in some non-food plants, and is therefore a preferred substrate for use in microbial fermentations. It is a reserve polysaccharide in the *Asteraceae* family, which includes chicory, dahlia, and Jerusalem artichoke (JAT), whichgrow in dry and infertile soils, and are known as C4-plants that are capable of active carbon dioxide fixation [20,21]. Derived from wild and adaptable plants, inulin flours or extracts are relatively cheap. For example, the retail price range for China chicory is between USD 0.38 and USD 0.76 per kilogram [22]. Therefore, inulin-containing materials are considered a cheap and abundant renewable feedstock for bioprocessing. They are already widely used in the biotechnological industrial production of inulinases [23,24], fructose and high fructose syrup [25,26], prebiotic fructooligosaccharides, single-cell protein, single-cell oil, organic acids such as citric, lactic, and gluconic acid, and bioethanol [27]. The bioprocesses for the production of acetone, butanol, and 2,3-butanediol are still under development [13,28].

However, the cost of microbial conversion to 2,3-BD increases with the necessary pretreatment steps, as all known biotechnologies to date carry out the inulin fermentation after enzymatic, acid, or combined acid-autoclaving hydrolysis to break down the inulin to fructose [29,30,31,32]. Currently, a significant amount of research concentrates on the efficient microbial production of 2,3-BD from inulin, but strains capable of simultaneous saccharification and fermentation (SSF) of inulin are rare [33,34,35].

Recently, we reported the isolation of such a strain, *B. lichenifomis* 24, which is a potent overproducer of 2,3-BD from soluble inulin without the need for preliminary hydrolysis. Although the strain did not degrade non-soluble Highly Dispersible inulin (Frutafit^®^ HD), it produced at rates as high as 67.5 g/L 2,3-BD by direct fermentation of soluble inulin Frutafit^®^ CLR [36]. However, increasingly large amounts of EPS formed when the processes were carried out at pH values above 6.00. Thus, the culture medium became thick and viscous, while the process at a higher pH than 6.25 was associated with ineffective aeration and the technical impossibility of conducting the fermentation. Consequently, the optimal pH for 2,3-BD production by *B. licheniformis* 24 (BL24) was never reached. When examining the acting enzymes, it was determined that at pH 6.25, the gene expression of *sacB* increased up to 197-fold compared to the process at pH 5.25 [36]. The enzyme sacB of *B. licheniformis* (E.C. 2.4.1.10, GH68) has dual hydrolase and transglycosylase activity. It is involved in the hydrolysis of inulin, but it is also responsible for the synthesis of levan in large quantities [37,38]. Therefore, levan synthesis can only be avoided by developing an engineered variant of the producer strain in which the levansucrase is blocked.

The present study aimed to knock out the *sacB* gene in BL24, investigate the effect of that modification on the total EPS synthesis and gene expression, and perform the process for maximal 2,3-BD production.

## 2. Results

### 2.1. Inactivation of sacB Gene in BL24

**Figure 1:**
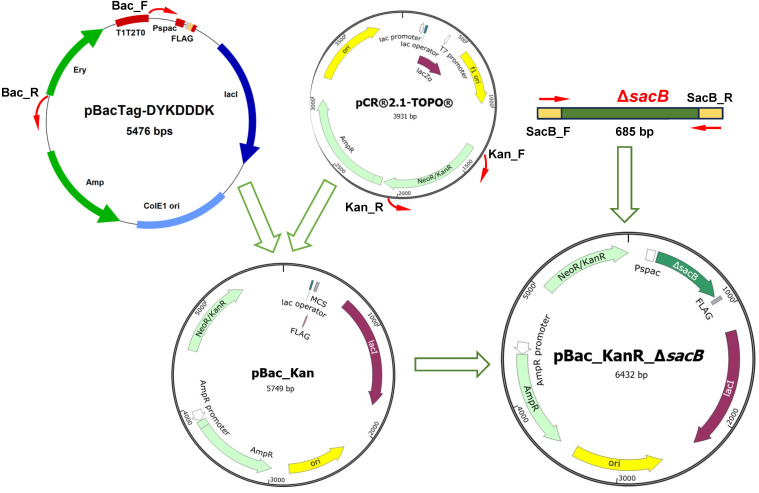
Cloning scheme. To eliminate the EryR gene and the adjacent terminator region, part of the pBac-TagDYKDDDK vector was amplified with a Bac_F and Bac_R primer pair, after which the resulting fragment (4738 bp) was ligated to KanR derived from the pCR^®^2.1-TOPO vector. The obtained construct pBac_Kan (5749 bp) was used for Gibson Assembly^®^ with the truncated *sacB* gene of BL24, resulting in the final pBac_KanR_Δ*sacB*. The sites and the direction of amplification are indicated with red arrows.

The purposeful inactivation of the *sacB* gene, encoding levan-producing levansucrase in BL24, was performed by insertional inactivation of a shuttle vector. Homologous recombination of a disrupted *sacB* and the reporter gene with the chromosome of BL24 was achieved in several cloning steps. The backbone of the construct was the integrative vector pBacTag-DYKDDDK (Figure 1). However, the selectable marker of the vector for erythromycin (EryR) appeared unsuitable because BL24 is resistant to erythromycin up to a concentration of 300 μg/mL. Since the host strain is susceptible to kanamycin at every concentration, aminoglycoside phosphotransferase from transposon Tn5, which confers resistance to kanamycin and neomycin (KanR/NeoR), was chosen as an appropriate reporter gene. Using the primer pairs Bac_F and Bac_R, the vector backbone was amplified with the excision of EryR and the T1T2T0 terminator sequence. The obtained partial version of pBacTag-DYKDDDK (4740 bp) was ligated by Gibson Assembly^®^ to the fragment (1009 bp) which contained KanR with its promoter and which was previously PCR-amplified from the pCR^®^2.1-TOPO vector as a template. The resulting shuttle vector, pBac_KanR (5749 bp), was linearized with KpnI, amplified, and assembled with the truncated and mutated Δ*sacB* (685 bp) obtained as a PCR product with template BL24 chromosomal DNA. The final construct, pBac_KanR_Δ*sacB* (6432 bp), was used to transform BL24.

After the transformation of electrocompetent *B. licheniformis* strain 24 and selection in the presence of 20 μg/mL kanamycin, 1.3 × 10^2^ transformants were obtained. These were screened for levan production by cultivation in Luria–Bertani (LB) medium with 50 g/L sucrose, followed by extraction and measurement of the total EPS. The clones that did not form EPS from fructose were further analyzed. The presence of the integrated construct was confirmed by PCR targeting Δ*sacB* and the reporter kanamycin gene, as well as sequencing of the products.

### 2.2. EPS Synthesis by BLΔsacB from Sucrose and Monosaccharides

**Figure 2:**
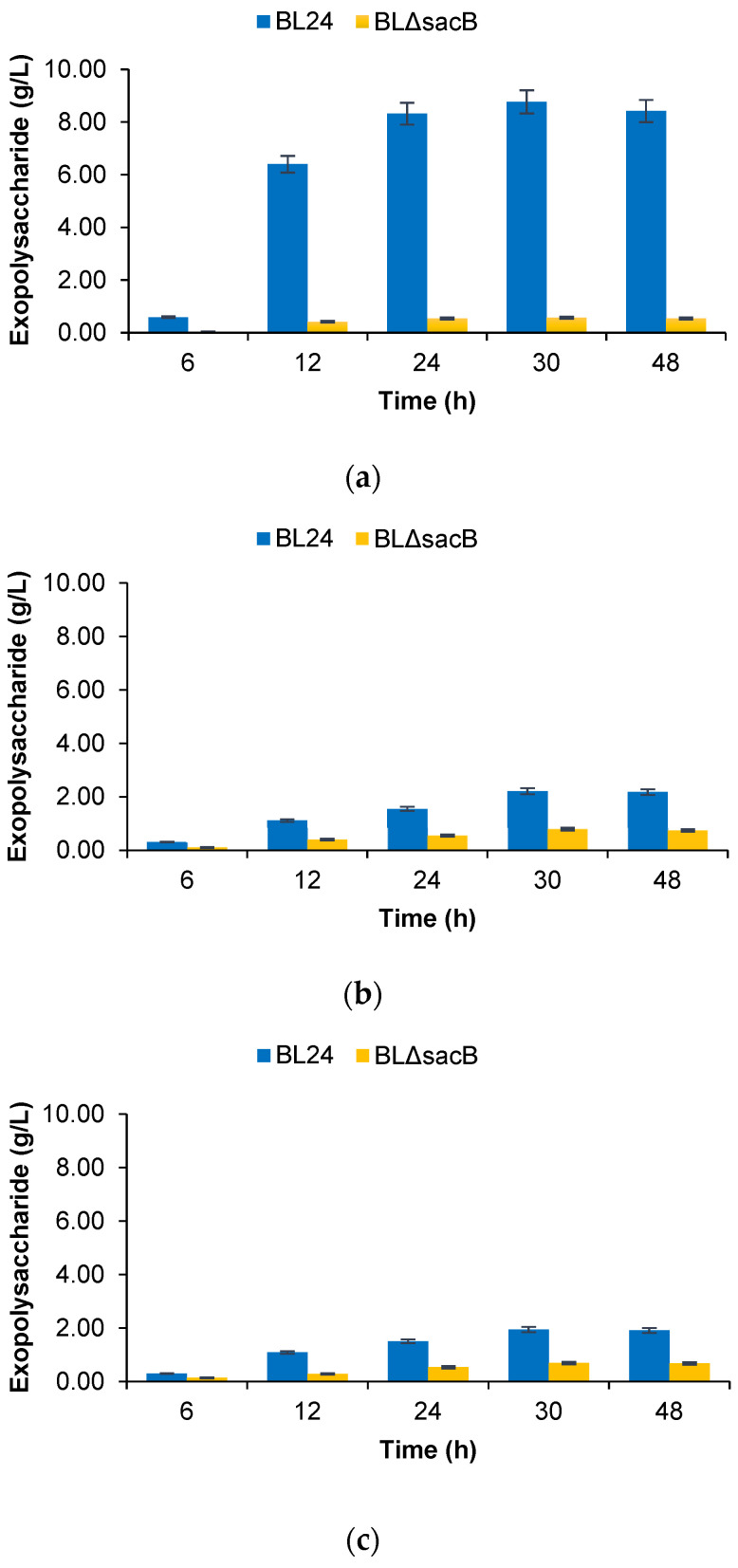
Time profiles of exopolysaccharides (EPS) formation by BL24 and its engineered variant, BLΔ*sacB*, during the course of flask-batch fermentation processes with a 50 g/L concentration of different sugars on a rotary shaker at 37 °C and 200 rpm for 48 h. (**a**) Sucrose; (**b**) Glucose; (**c**) Fructose.

The total EPS production by the engineered BLΔ*sacB* was compared with those of BL24 during flask-batch fermentation without pH control in LB broth supplemented with 50 g/L sucrose, glucose, or fructose. In the medium containing sucrose, the wild type formed large amounts of EPS, reaching 8.78 g/L at the 30th hour of fermentation, while the *sacB*-knockout variant BLΔ*sacB* formed only 0.57 g/L, or a 15-fold lower amount (Figure 2a). From glucose and fructose, the wild type formed about 2 g/L of EPS, while BL*ΔsacB* formed between 0.7 and 0.8 g/L, or about 2.5 times less (Figure 2b).

### 2.3. Effect of pH on Inulin Conversion to 2,3-BD by the Engineered BLΔsacB

Among process parameters, pH maintenance is crucial for the successful conversion of inulin to 2,3-BD. With increasing pH, 2,3-BD increased; however, it increased alongside a high accumulation of EPS, leading to uncontrollable thickening of the culture broth. Thus, the highest pH at which inulin fermentation by native BL24 can occur is pH 6.25. In contrast, with the engineered variant BLΔ*sacB*, several processes at pH maintained at 6.25, 6.50, and 6.75 were carried out, as there was no significant production of EPS impeding the fermentation. Table 1 presents the obtained metabolites (2,3-BD, acetoin, and byproducts) at the moment of the highest production of 2,3-BD.

For a reliable assessment of the qualities of the mutant BLΔ*sacB* as a producer, a process with 200 g/L soluble inulin at pH 6.25 was carried out. The obtained results and fermentation kinetics were compared with those of the wild-type BL24 as reported by Tsigoriyna et al. [36]. BLΔ*sacB* formed a higher amount of 2,3-BD compared to the wild type (75.5 g/L vs. 67.5 g/L), accompanied by negligible EPS formation. Byproducts remained within close limits, with slightly more glycerol produced (14.1 g/L by BLΔsacB vs. 9.8 g/L by BL24).

**Figure 3:**
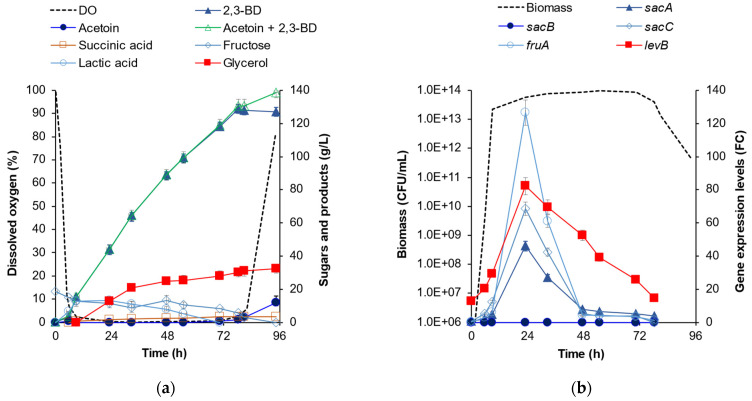
Production of 2,3-butanediol by BLΔ*sacB* from 300 g/L soluble chicory flour Frutafit^®^ CLR at pH 6.50. (**a**) Time profile of 2,3-BD and byproducts formation, fructose and dissolved oxygen (DO); (**b**) Gene overexpression presented as a fold-change compared to the rate observed at 0 h of the process carried out at pH 5.25 [36]. The fermentation was performed in a bioreactor at 37 °C, with agitation at 500 rpm and aeration at 3.68 vvm.

Figure 3a shows the kinetics of the process conducted at pH 6.50 with the mutant strain. Converting 300 g/L soluble chicory flour, BLΔ*sacB* formed 128.7 g/L 2,3-BD and 1.8 g/L acetoin. The main byproduct was glycerol (30.2 g/L), followed by a small amount of succinic acid (3.3 g/L). RT-qPCR analysis showed that the genes involved in the hydrolysis of inulin, *sacA*, *sacC*, *fruA*, and *levB,* significantly increased their expression levels, reaching peak values at an mRNA level on the 24th hour (after the exponential phase), then dropping to baseline expression at 48 h (Figure 3b).

At pH 6.75, BLΔ*sacB* reached 96.4 g/L 2,3-BD and 2.1 g/L acetoin, which was some decrease in the amount of target product compared to the process at pH 6.50. This was attributed to a high concentration of lactic acid that was formed in this process (44.8 g/L), and thus, LA became the main byproduct, significantly ahead of glycerol (10.9 g/L). Since at higher pH*,* the metabolic flux was directed towards LA formation, this result may suggest that the pH optimum for 2,3-BD synthesis was at pH 6.50. For comparison, at pH 6.50, the lactic acid was a temporary product only (Figure 3a). Also, in the same process (pH 6.50), LA reached its maximum of 13.1 g/L at the 24th hour and thereafter was completely assimilated to the 70th hour of the fermentation. At pH 6.75, the visual viscosity of the culture slightly increased notwithstanding the lack of *sacB* expression, thus suggesting that BLΔ*sacB* continued to form EPS different from levan using the glucose and fructose derived from inulin hydrolysis.

### 2.4. Reverse Transcription Real-Time PCR (RT-qPCR)

**Figure 4:**
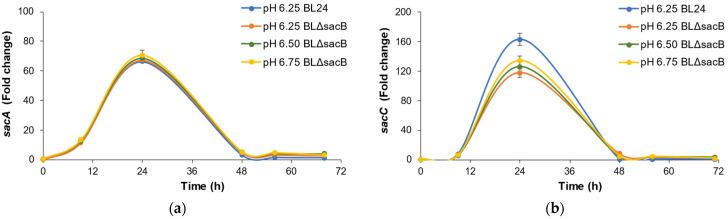

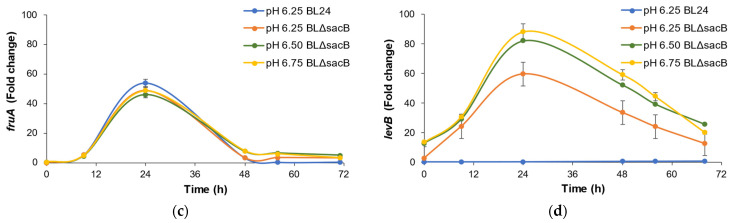
Fold change (FC) in gene expression of *sacA*, *sacC*, *fruA*, and *levB* genes involved in inulin hydrolysis during processes of Frutafit^®^ CLR conversion to 2,3-butanediol by engineered BLΔ*sacB* with pH maintained at the values indicated. FC was calculated vs. 0 h at pH 5.25 of BL24 [36]. (**a**) *sacA*; (**b**) *sacC*; (**c**) *fruA*; (**d**) *levB*.

During fermentation, four genes involved in inulin hydrolysis significantly changed their expression levels at different pH: *sacA*, *sacC*, *fruA*, and *levB* (Figure 4).

The lack of expression of *sacB* proves its successful disruption. Compared to the wild-type BL24, *sacA*, *sacC*, and *fruA* attained their upregulated expression at pH 6.25 vs. pH 5.25, conforming to the results shown previously by Tsigoriyna et al. [36]. However, the behavior of *levB* was completely different (Figure 4d). Its expression increased between 60- and 88-fold, depending on the pH of the processes with BLΔ*sacB*, while it remained unaffected in the wild-type BL24. At the 48th h, all gene overexpression (except that of *levB*) entirely declined. The upregulation of *levB* remained high through to the end of the processes, with over 20-fold overexpression in BLΔ*sacB* until the 72nd h.

## 3. Discussion

A decade ago, a pilot microbial plant in the UK for low-impact production of 2,3-BD with a capacity of 30,000 tons per year was reported to reduce CO_2_ emissions by more than 50% [39], thus revealing the significant effect of substituting fermentation for chemical synthesis. The most widely used substrates in industrial biotechnologies for 2,3-BD synthesis are glucose, starch, molasses, and glycerol. According to a techno-economic evaluation performed by Koutinas et al. [11], glycerol is the most profitable, followed by molasses. Since the cost of the raw materials that serve as a carbon source accounts for more than 30% of the total production costs of fermentation, low-price substrates and efficient strains are required for 2,3-BD production [32]. Inulin is identified as an inexpensive, nonfood, renewable source, as it is the third most abundant reserve polysaccharide on the planet (after cellulose and starch). Currently, the price of inulin has leveled off with that of molasses, ranging between 140 and 190 USD per ton [40]. Due to its increasing importance in microbial production on an industrial scale, the global inulin market is expected to reach 1.86 billion USD by the end of 2024. For the period from 2024 to 2029, the demand for inulin is expected to grow at a Compound Annual Growth Rate (CAGR) of 6.55%, with the fastest rate in the Asia-Pacific region and the largest market in Europe, with the global inulin market ultimately reaching 2.56 billion USD by 2029 [41]. On the other hand, the plants from which inulin can be extracted have good productivity (15–50 tons per hectare), and resistance to low temperatures and disease; the time to first grazing is typically 55–85 days after planting [19].

However, a hitherto unsolved problem of industrial fermentations involving *B. licheniformis*, regardless of the target product, is the thickening of the culture due to simultaneous EPS synthesis and uncontrollable foaming during the process [42]. The reason is that the natural strains of *B. licheniformis* simultaneously produce several exopolymers (polyglutamic acid, levan, and heteropolysaccharides) [43], polyols (glycerol and 2,3-BD), and biosurfactants [44]. Levan is the most typical EPS produced by *B. licheniformis* in quantities exceeding 50 g/L (Table 2). Accumulated in high amounts, it increases the viscosity of the fermentation broth and alters some of the process parameters, like aeration and agitation.

EPS production by *B. licheniformis* depends on the substrate and its quantity [49,50,51], the fermentation mode (batch or fed-batch) [52,53], and the process parameters, with pH being of key importance [54]. The monosaccharides glucose and fructose contribute to the formation of hetero-EPS, most often consisting of glucose, galactose, and mannose [39,52]. High sucrose concentrations, such as in molasses, provoke levan formation by levansucrase [44,45,46,47]. However, during flask-batch processes, regardless of the initial amount of the mono-sugar, EPS synthesis is limited due to the mixed-acid type of fermentation, with lactic and succinic acid among the byproducts. In these processes, the pH of the medium has been observed to spontaneously drop to below pH 5.25, which does not aid the elevated formation of EPS. On the other hand, when the pH maintains a relatively high level (pH 6.0–pH 7.0), it accelerates the formation of both hetero-EPS and levan.

Until now, these phenomena have not been considered in the process of inulin conversion to 2,3-BD by *B. licheniformis*. The Frutafit^®^ CLR chicory flour used in our study contains 80.6% inulin and 19.4% sugars obtained during chicory root processing, of which 7.9% is fructose, 1.5% glucose, and 10% sucrose. Moreover, inulin chains in this substrate have an average degree of polymerization (DP) between 7 and 9, as each of them ends with a sucrose residue, which is an additional substrate for the levansucrase. During the fermentation by the natural strain BL24 at pH 6.25, 197-fold overexpression of *sacB* supported the accumulation of levan in large amounts; therefore, a key strategy to solve this issue is knocking out the *sacB* gene in the wild-type producer.

Some authors focus on the potential benefits of the simultaneous synthesis of levan and 2,3-BD in a mixture [44], but others highlight the difficulties inherent to the subsequent purification steps of either target product and develop engineered strains for less EPS and reduced foaming [55,56]. Mining in the *B. licheniformis* genome revealed that hetero-EPS are produced by a cascade pathway encoded by the operon *epsA-O*, which comprises 16 *eps* genes [57,58]. To reduce EPS formation for efficient alkaline phosphatase production, Zhou et al. [56] knocked out the *eps* cluster in *B. licheniformis* 2709 and disrupted the *lchA-C* genes to diminish the foaming. Applying the same approach, Song et al. [44] accomplished *epsAB* knockout and obtained 88 g/L 2,3-BD together with 70 g/L levan from sucrose.

In our research, the strategy to reduce the total amount of EPS was to inactivate the *sacB* gene encoding levansucrase. This was accomplished by the construction of BLΔ*sacB,* an engineered variant of BL24 containing a disrupted *sacB* gene. The batch fermentation of mono sugars and sucrose showed that *sacB* inactivation resulted in a large reduction in EPS formation from sucrose (more than 15-fold) and a 2.5 times reduction in EPS from glucose and fructose by the recombinant clone compared to the wild type. The overexpression of other genes involved in inulin hydrolysis, *sacA*, *sacC*, and *fruA* encoding sucrases (invertases, EC 3.2.1.26), remained as high as that of the wild type. The *levB* gene encoding levanase (EC 3.2.1.65) showed gradual upregulation in BLΔ*sacB* in a pH-dependent manner and reached its maximum overexpression at pH 6.75. The ~88-fold difference in expression compared to the wild type is possibly due to the changed genomic context after the vector’s integration.

Recently, considerable progress has been made in attempts to engage GRAS (Generally Regarded as Safe) bacteria and to utilize different types of inulin for 2,3-BD synthesis [11,30,31,32]. From acid- or enzyme-hydrolyzed JAT extract by *Paenibacillus polymyxa* ATCC 12321 were obtained 44.0 g/L 2,3-BD [30]; by *P. polymyxa* ZJ-9—37.1 g/L [31]; by *Bacillus* sp. BRC1—28.6 g/L [35], and by *K. pneumoniae* CICC 10011—84.0 g/L [35]. Notably, the highest titer of 2,3-BD was achieved by the type of strain *B. licheniformis* ATCC 14580^T^ from chicory inulin. Li et al. (2014) performed one-pot saccharification and fermentation with hydrolysis supported by the external additions of recombinant inulinase (SacC), whichyielded 103.0 g/L 2,3-BD in 30 h with a productivity of 3.4 g/L h [32].

In this study, an engineered variant of BL24, distinguished by its ability to convert directly soluble inulin (without the need for preliminary hydrolysis), was developed via *sacB* knockout. The genetically improved BLΔ*sacB* achieved a particularly high titer of 128.7 g/L 2,3-BD. The mutant converted 300 g/L soluble chicory flour in an SSF process and produced the target metabolite with a yield of 0.429 g/g, which is 85.8% of the theoretical yield. The approach of EPS total decrease by *sacB* gene knockout was applied for the first time and apparently is highly effective. By solving the problem of undesired levan accumulation, this study opens the prospect of industrial production of 2,3-BD from inulin-rich substrates by the use of an engineered GRAS producer.

## 4. Materials and Methods

### 4.1. Bacterial Strains, Media, and Cultivation Conditions

The wild-type *B. licheniformis* strain 24 is stored in the collection of the Institute of Microbiology, Bulgarian Academy of Sciences, and was identified previously by 16S rRNA gene sequencing (GenBank accession no. MK461938.1) [59].

*E. coli* HST08 (STELLAR^TM^) competent cells were used in vector constructions and gene cloning (Table 3). The strain was cultivated in LB medium, broth, or supplemented with 15 g/L agar (Alfa Aesar GmbH & Co. KG; Karlsruhe, Germany), and kanamycin with final concentrations of 50 μg/mL (AppliChem GmbH, Darmstadt, Germany).

BL24 was maintained on slant LB-agar tubes at 4 °C or as a frozen liquid culture supplemented with 20% glycerol at −70 °C.

Fermentations for 2,3-BD production and EPS synthesis estimation were carried out in the FM (fermentation medium) optimized by Tsigoriyna et al. [18], containing (g/L): sugars (glucose, fructose, sucrose), 50; or CFP (chicory flour powder), 200 or 300; tryptone, 6.41; yeast extract, 13.38; ammonium acetate, 2.5; (NH_4_)_2_SO_4_, 1; KH_2_PO_4_, 3.5; K_2_HPO_4_, 4.2; MgSO_4_, 0.32; CoCl_2_·6H_2_O, 0.09; microelements solution, 3 mL per liter, containing (g/L): FeSO_4_, 0.4, H_3_BO_3_, 0.8; CuSO_4_·5H_2_O, 0.04; NaMoO_4_·2H_2_O, 0.04; MnCl_2_·4H_2_O, 5.0; ZnSO_4_·7H_2_O, 0.1; Co(NO_3_)_2_·6H_2_O, 0.08; CaCl_2_·2H_2_O, 1.0; Biotin, 0.01. All chemicals were purchased from Sigma-Aldrich Co. (Saint Louis, MO, USA).

Soluble chicory flour Frutafit^®^ CLR (Sensus B.V., Roosendaal, The Netherlands) was used as a CFP substrate.

Flask-baches were performed as BL24 was cultured in 500 mL Erlenmeyer flasks containing 100 mL of fermentation medium, as the inoculum was a 1% overnight culture. Flask cultivation was on a rotary shaker at 37 °C and 200 rpm.

Batch processes with pH and aeration control were carried out in a 1 L Biostat^®^ A Plus stirred bioreactor (Sartorius Stedim Biotech, Gottingen, Germany), with an additional air pump, rotameter, and vessel for possible excess foam removal. The FM medium described above was used. The process parameters were maintained at optimal values for glucose conversion: temperature 37.8 °C and aeration 3.68 vvm [18]. The pH was maintained at different constant values by adding 6M NaOH or 5M HCl. The added inoculum was 10% (overnight culture of BL24 with OD_600_ = 2.0).

### 4.2. DNA and RNA Isolation, PCR, and Gibson Assembly Cloning

Total DNA and RNA from samples taken at different hours were isolated with a GeneMATRIX Bacterial & Yeast Genomic DNA Purification Kit and a GeneMATRIX Universal Purification Kit, respectively, according to the instructions of the manufacturer (EURx, Gdansk, Poland).

PCR for fragments shorter than 4 kb was performed with TaKaRa Taq Version 2.0 (Clontech Laboratories, Inc., A Takara Bio Company, Mountain View, CA, USA) in 25 μL reaction volume with 50 ng DNA template and 0.4 μM primers (Table 4). Initial denaturation was set for 1 min at 94 °C, while the hot start was ensured by 10 s at 98 °C each cycle. Elongation time was 1 min per kilobase for each cycle and 5 min final after 35 cycles of amplification. Fragments longer than 4 kb were amplified with TaKaRa LA Taq^®^ DNA Polymerase (Clontech Laboratories, Inc., A Takara Bio Company, Mountain View, CA, USA) in the same reaction volume and with the same amount of template. The reaction mixture also contained freshly added 2.5 mM MgCl_2_, 0.4 mM from each of the four dNTPs, 0.5 μM of each primer, and 1U of the polymerase. Elongation time was 52 s per kilobase for each cycle and 10 min final after 35 cycles of amplification. All PCR reactions were performed in MultiGene OptiMax Thermal Cycler (Labnet International, Inc., Edison, NJ, USA).

PCR products were visualized on agarose gel (1%) with the addition of fluorescent dye SimplySafe (EURx, Gdansk, Poland) diluted 1:20,000. Sequencing was performed courtesy of Macrogen Inc. (Amsterdam, The Netherlands).

An NEBuilder^®^ HiFi DNA Assembly Cloning Kit (New England Biolabs, Ipswich, MA, USA), an advanced Gibson Assembly cloning system, was used to obtain the recombinant constructs. Ligation reactions were pipetted on ice and consisted of 10 μL NEBuilder HiFi DNA Assembly Master Mix, 200 ng vector, and an amount insert calculated to be in 1:1 molar ratio, and sterile H_2_O to 20 μL. Incubation was conducted at 50 °C for 1 h. One-fourth of the reaction was used for the transformation of the NEB 5-alpha competent cells provided with the kit. The mixture of 50 μL competent cells and 5 μL ligation reaction was incubated for 30 min on ice and heat shocked for 50 s at 42 °C. After a brief chill on ice, 950 μL warmed SOC (Super Optimal broth with Catabolite repression; LB with 2% tryptone, 10 mM NaCl, 2.5 mM KCl, 10 mM MgCl_2_ and 20 mM glucose) was added and the cells were incubated for 1 h at 37 °C and 160 rpm. Transformants were selected after plating 100 μL of the cells on LB-Agar dishes with 50 μg/mL kanamycin and overnight incubation at 37 °C.

### 4.3. Transformation of E. coli and BL24

Transformation of *E. coli* STELLAR^TM^ competent cells (Clontech Laboratories, Inc., A Takara Bio Company, Mountain View, CA, USA) was carried out following Protocol PT5055-2 from the manufacturer. Briefly, 50 μL cells were thawed on ice, mixed with the construct to be transformed, incubated on ice for 30 min, heat shocked for 45 s at 42 °C, and returned to resto on ice for 1–2 min. Warmed SOC medium was added to the final volume of 500 μL and the cells were incubated for 1h at 37 °C and 160 rpm. Cells were then plated on standard Petri dishes with 50 μg/mL kanamycin for the selection of transformants.

BL24 was transformed with a slightly modified version of the high-osmolarity electroporation protocol by Xue et al. [59]. An overnight culture in standard LB medium (1% tryptone, 0.5% yeast extract, 0.5% NaCl), diluted 16 times in LB medium with 0.5 M sorbitol in a 500 mL Erlenmeyer flask, was grown until it reached an OD_600_ of 0.9. The cells were chilled on ice for 10 min and then washed four times with ice-cold electroporation medium (0.5 M sorbitol, 0.5 M mannitol, 10% glycerol). After the last centrifugation (3350 g/10 min/4 °C), the competent cells were resuspended in a final volume of 625 µL electroporation medium, separated into aliquots of 60 µL, and stored at −70 °C. Electroporation was performed in cuvettes with a 0.1 cm electrode gap on a MicroPulser electroporator (BioRad Laboratories, Hercules, CA, USA) with a pulse of 2.1 kV applied for the optimal time of 4–5 ms. Recovery medium (1 mL, 0.5 M sorbitol, and 0.38 M mannitol in LB) was added immediately afterward. The culture was transferred into 15 mL glass tubes and incubated for 3 h at 37 °C. Selection of transformants was obtained on LB-agar plates with 50 μg/mL kanamycin after overnight incubation at 37 °C. The stability of the mutant strain was confirmed by plating it for five passages without antibiotic selective pressure.

### 4.4. RT-qPCR

Reverse transcription (RT) was accomplished with the NG dART RT Mix (EURx, Gdansk, Poland). The 20 μL reactions contained 1 μg total RNA and 200 ng random hexamer primers and were subjected to the following program: 10 min at 25 °C for primer hybridization, 50 min at 50 °C for the reverse transcription itself, and 5 min at 85 °C for the inactivation of the enzyme. Before the reverse transcription, all RNA samples were subjected to DNase I (5U) treatment in a buffer with 25 mM MgCl_2_ (EURx, Gdansk, Poland) for 30 min at 37 °C. Enzyme inactivation was achieved for 10 min at 65 °C with the addition of 20 mM EDTA. RT-qPCR was performed with SsoFast™ EvaGreen^®^ Supermix with Low ROX (Bio-Rad, Hercules, CA, USA) in a Corbett Research RG-6000 Real-Time PCR Thermocycler (Qiagen, Germantown, MD, USA). Primer pairs were designed to amplify fragments from 62 to 125 bp within *sacA*, *sacB*, *sacC*, *levB*, *fruA*, and the control 16S rRNA gene (Table 5).

The optimal annealing temperature was determined to be 60 °C. Each reaction of 20 μL contained 40 ng cDNA as a template and 500 nM primers. 16S rRNA was used as an internal control for each sample in each run. The beginning (0 h) at the lowest pH (5.2) was used as a basis for comparison. Relative expression was calculated by the ΔΔCt method as described previously [36]. The presented results are the mean values of three independent fermentation experiments, each tested by three independent RT-PCR trials. The standard deviation was 6%.

### 4.5. Analytical Methods

The cell growth of the inoculum cultures was monitored by measuring the optical density (OD) at wavelength 600 using a UV/VIS Spectrophotometer (Thermo Scientific Inc., Waltham, MA, USA).

The viable cell counts (CFU, colony-forming units, per mL) were estimated as decimal dilutions of samples plated on LB-agar plates.

RNA concentrations and purity (Abs_260_/Abs_280_ ratio) were determined using Quawell UV Spectrophotometer Q3000 (Quawell Technology, Inc., San Jose, CA, USA).

The concentrations of the obtained 2,3-BD, acetoin, glycerol, lactic, and succinic acids were analyzed with a YL Instrument 9300 HPLC System (YL Instrument Co., Ltd., Anyang, Republic of Korea) equipped with an RI detector (YL 9170), using HPLC column Aminex HPX-87H at 65 °C with a mobile phase of 5 mM of H_2_SO_4_ at a flow rate of 0.6 mL/min (BioRad Laboratories, Hercules, CA, USA). For glucose, fructose, and sucrose quantification, a column HPX-87C (BioRad Laboratories, Hercules, CA, USA) at 85 °C was used. As a mobile phase, water with a flow rate of 0.6 mL/min was used.

All EPSs formed from glucose, fructose, and sucrose were extracted by the following procedure. Crude EPS fractions were isolated from the fermentation medium after initial centrifugation at 6000× *g* for 30 min to remove the biomass. The supernatant was deproteinated by incubation with 14% trichloroacetic acid (Merck KGaA, Darmstadt, Germany) in a rotary shaker (90 rpm) at 37 °C for 40 min. The sample was then centrifuged at 10,000× *g* for 10 min, at 4 °C, to remove the denatured proteins. The supernatant (crude EPS) was precipitated against three volumes of ice-cold ethanol (96%) and incubated at –18 °C overnight. The EPSs were harvested by centrifugation at 10,000× *g* for 20 min, washed twice with 50% ethanol, and the pellet was air-dried and dissolved in sterile dd H_2_O. After overnight dialysis at 4 °C, the sample was dried in a desiccator and stored at 4 °C. Bradford assay showed that all EPSs did not contain any amounts of residual protein. The carbohydrate content in each EPS was tested with the phenol-sulfuric acid colorimetric method of Dubois [60].

## 5. Conclusions

The present study reports a record amount of 2,3-BD produced from inulin involving the engineered BLΔ*sacB*, a variant of the natural isolate BL24. By disrupting a single gene, *sacB,* encoding levansucrase, the mutant BLΔ*sacB* produced 15-fold less levan from sucrose than the wild type. Thus, when the fermentation was carried out at the optimum process pH of 6.50, 300 g/L soluble chicory flour was directly converted to 128.7 g/L 2,3-BD, with a productivity of 1.65 g/L/h and a yield of 0.43 g/g, which is close to the theoretical maximum. The two-fold increase in 2,3-BD titer obtained from the engineered strain compared to the wild type reveals the great potential of the mutation in the *sacB* gene to solve the problem of undesired levan accumulation by *B. licheniformis*.

## Figures and Tables

**Table 1:** Batch fermentation of soluble chicory flour Frutafit^®^ CLR to 2,3-butanediol at different pH levels by BL24 and its engineered variant BLΔ*sacB*. The average final concentrations of the products obtained in three independent experiments are shown, with a standard deviation of 3%. All fermentations were conducted in a 1 L stirred fermenter at a temperature of 37.8 °C, aeration 3.68 vvm, and agitation 500 rpm. The fold change was calculated vs. 0 h at pH 5.25 for BL24 [36]. The comparative ΔΔCt method was used for the estimation of the relative abundance of each gene on the mRNA level. The 16S rRNA gene was used for normalization.

Strain	Conditions		Products					Maximum Gene Expression Levels				
	CFP ^1^ (g/L)	pH	2,3-BD (g/L)	Acetoin (g/L)	Glycerol (g/L)	LA ^2^ (g/L)	SA ^3^ (g/L)	*sacA* (FC ^4^)	*sacB* (FC ^4^)	*sacC* (FC ^4^)	*fruA* (FC ^4^)	*levB* (FC ^4^)
BL24 ^5^	200	6.25	67.5 ± 3.5	0.3 ± 0.3	9.8 ± 1.4	ND	1.3 ± 0.1	66.26 ± 2.1	196.72 ± 6.1	163.14 ± 5.8	53.82 ± 2.4	0.40 ± 0.1
BLΔ*sacB*	200	6.25	75.5 ± 2.3	1.3 ± 1.2	14.1 ± 1.6	ND	0.5 ± 0.1	64.71 ± 2.3	ND	118.16 ± 5.4	49.13 ± 2.1	59.73 ± 1.9
BLΔ*sacB*	300	6.50	128.7 ± 4.1	1.8 ± 1.1	30.2 ± 2.5	ND	3.3 ± 0.2	68.45 ± 2.8	ND	126.64 ± 5.7	46.03 ± 2.3	82.16 ± 5.2
BLΔ*sacB*	300	6.75	96.4 ± 4.7	2.1 ± 2.0	10.9 ± 2.3	44.8 ± 2.8	2.0 ± 0.2	70.40 ± 3.2	ND	134.24 ± 5.8	48.79 ± 2.3	88.19 ± 5.3

^1^ CFP, Chicory flour powder; ^2^ LA, Lactic acid; ^3^ SA, Succinic acid; ^4^ FC, Fold change; ^5^ Previously described process with BL24 performed under the same conditions by Tsigoriyna et al. [36] was used for comparison. ND, not determined.

**Table 2:** EPS produced by *B. licheniformis* strains.

Strain	Substrate	EPS	Description	Reference
*B. licheniformis*	Sucrose	71 g/L	Levan, Δ*epsAB*	[44]
*B. licheniformis* NS032	Sucrose	53.20 g/L	Levan, optimum pH 7.2	[45]
*B. licheniformis* 8-37-0-1	Sucrose	47.45 g/L	Levan, optimum pH 6.5–7.0	[46]
*B. licheniformis* ANT 179	Sugarcane juice	50.25 g/L	Levan, optimum pH 7.0	[47]
BL24	Glucose (fed-batch)	12.61 g/L	EPS of galactose, glucose, and mannose in ratio 54/39/7; pH 6.23	[48]
BL24	Fructose (fed-batch)	7.03 g/L	EPS of glucose, mannose, and galactose in ratio 51/30/19; pH 6.23	[48]

**Table 3:** Bacterial strains and plasmids were used in this study.

Strain/Plasmid	Description and Use	Source or Reference
BL24	Natural isolate from a soil sample taken near Yantra River’s bed near Veliko Tarnovo, Bulgaria (43°04′52.46″ N 25°37′44.54″ E). Used as a host for *sacB* gene disruption.	[18,36]
*E. coli* STELLAR^TM^	*F-*, *endA1*, *supE44*, *thi-1*, *recA1*, *relA1*, *gyrA96*, *phoA*, *Φ80d lacZ*Δ *M15*, Δ*(lacZYA-argF) U169*, Δ*(mrr-hsdRMS-mcrBC)*, Δ*mcrA*, *λ-.* Used as a host in cloning procedures.	Takara Bio Company (Mountain View, CA, USA)
pBacTag-DYKDDDDK	*B. subtilis* chromosomal integration vector; EryR, AmpR, Epitope FLAG tag. Used for Δ*sacB* disruption cassette construction.	MoBiTec GmbH, Goettingen, Germany
pCR^®^2.1-TOPO^®^	*E. coli* TOPO-TA cloning vector. Used as a source of the KanR (NeoR) gene.	Thermo Fisher Scientific Inc., Waltham, MA, USA
BLΔ*sacB*	A mutant of BL24 containing *sacB* gene knockout. Used for 2,3-BD production.	This study
pBac_Kan	Chromosomal integration vector; KanR, AmpR. Used for cloning of Δ*sacB* PCR fragment.	This study
pBac_Kan_Δ*sacB*	Δ*sacB*—containing integrative construct. Used for Δ*sacB* knockout in BL24 chromosomes.	This study

**Table 4:** Nucleotide sequences of the primers used for gene amplification and/or sequencing. Underlined bases = inserted RBS (Ribosome-binding site). Bases in bold = inserted stop codons. Bases in italics = tails for cloning with Gibson Assembly^®^.

Primer	Sequence (5′–3′)	PCR Product	Tm (°C)	Molecule Size (bp)
Bac_F	attctatgagtcgcttttgtaaatt	pBacTag_Δ*EryR*	63.8	4738
Bac_R	tgtaatcactccttcttaattacaa		62.4	
Kan_F	*gaaggagtgattaca*aaagagaaagcaggtagcttgc	KanR	65.2	1009
Kan_R	*agcgactcatagaat*tcagaagaactcgtcaagaaggcg		68.5	
BK_F	ggattataaagatgatgatgataaa	pBacTag_KanR	59.2	5747
BK_R	ggtaccctcgactctagat		63.4	
sac_F	*gatctagagtcgagggtacc*tac**taatag**caaggagaagactccctattc	Δ*sacB*	61.0	685
sac_R	*ctttataatccggcc*gaaaattccccgctttattctaag		62.0	

**Table 5:** Primers used in RT-qPCR experiments.

Primer	Sequence (5′–3′)	PCR Product (bp)	Position in Gene *
16S_F	gagtacgaccgcaaggttga	100	875–895
16S_R	cctggtaaggttcttcgcgt		975–955
sacA_RTF	aagagatcgccctcacgccgagcgactggttt	125	255–286
sacA_RTR	atttccctcgccgtctctgacattccccgtgt		379–348
sacB_RTF	caacagagcctactacgggggcagcaagaagt	117	861–892
sacB_RTR	tcgatgattccgagagcgccgttagccagcga		977–946
sacC_RTF	gccgctcgttgccatttatacgcaggaccgga	64	375–406
sacC_RTR	gctgtaggcgatgctttgcacttgttccccgc		438–407
levB_RTF	gcatactggacaggcagcttcaacggcaacga	121	784–815
levB_RTR	cgttcgtttcgccgtcctcaaatgtcacgccc		904–873
fruA_RTF	gggagtcagagatgccgacgaaagcagacgga	62	893–924
fruA_RTR	ttcacgcggcaaagttaatgccccgcaccatc		954–923

* Positions are according to the respective gene sequences *B. licheniformis* ATCC 14580^T^ (NCBI GenBank acc. no. CP034569.1).

## Data Availability

Nucleotide sequences are available in the NCBI GenBank. The strain BL24 and its engineered variant can be obtained from the authors on request.
